# Evaluation of inorganic phosphate solubilizing efficiency and multiple plant growth promoting properties of endophytic bacteria isolated from root nodules *Erythrina brucei*

**DOI:** 10.1186/s12866-022-02688-7

**Published:** 2022-11-19

**Authors:** Belay Berza, Jegan Sekar, Prabavathy Vaiyapuri, Marcela C. Pagano, Fassil Assefa

**Affiliations:** 1 Department of Biology, Debre Markos University, Debre Markos, Ethiopia; 2 Microbiology Laboratory, MS Swaminathan Research Foundation, Chennai, Tamil Nadu India; 3 Department of Biology, Federal University of Minas Gerais, Belo Horizonte, Brazil; 4 Department of Microbial, Cellular and Molecular Biology, Addis Ababa University, Addis Ababa, Ethiopia

**Keywords:** *Achromobacter*, *Acinetobacter*, *Bacillus*, *Gluconobacter*, Phytobeneficial traits, Phosphate mobilization

## Abstract

**Background:**

In soils, phosphorous (P) mostly exists in fixed/insoluble form and unavailable for plants use in soil solution, hence it is in scarcity. P is fixed in the form of aluminium, iron and manganese phosphates in acidic soils and calcium phosphate in alkaline soils. Phosphate solubilizing bacteria, the ecological engineers play a pivotal role in the mobilization of fixed forms of P by using different mechanisms. The objectives of this study were to evaluate inorganic phosphate solubilizing efficiency and other multiple plant growth promoting traits of *Erythrina brucei* root nodule endophytic bacteria and to investigate effects of the selected endophytic bacteria on the growth of wheat plant under phosphorous deficient sand culture at greenhouse conditions.

**Results:**

Among a total of 304 passenger endophytic bacteria, 119 (39%) exhibited tricalcium phosphate (TCP) solubilization; however, none of them were formed clear halos on solid medium supplemented with aluminum phosphate (Al-P) or iron phosphate (Fe–P). Among 119 isolates, 40% exhibited IAA production. The selected nine potential isolates also exhibited potentials of IAA, HCN, NH_3_ and/or hydrolytic enzymes production. All the selected isolates were potential solubilizers of the three inorganic phosphates (Al-P, Fe–P and TCP) included in liquid medium. The highest values of solubilized TCP were recorded by isolates AU4 and RG6 (*A. soli*), 108.96 mg L^−1^ and 107.48 mg L^−1^, respectively at sampling day3 and 120.36 mg L^−1^ and 112.82 mg L^−1^, respectively at day 6. The highest values of solubilized Al-P and Fe–P were recorded by isolate RG6, 102.14 mg L^−1^ and 96.07 mg L^−1^, respectively at sampling days 3 and 6, respectively. The highest IAA, 313.61 µg mL^−1^ was recorded by isolate DM17 (*Bacillus thuringiensis*). Inoculation of wheat with AU4, RG6 and RG5 (*Acinetobacter soli*) increased shoot length by 11, 17.4 and 14.6%, respectively compared to the negative control. Similarly, 76.9, 69.2 and 53.8% increment in shoot dry weight is recorded by inoculation with RG6, AU4 and RG5, respectively. These nine potential endophytic isolates are identified to *Gluconobacter cerinus* (4), *Acinetobacter soli (*3)*, Achromobacter xylosoxidans* (1) and *Bacillus thuringiensis* (1).

**Conclusion:**

AU4, RG6 and RG5 can be potential bio-inoculants candidates as low cost agricultural inputs in acidic and/or alkaline soils for sustainable crop production.

**Supplementary Information:**

The online version contains supplementary material available at 10.1186/s12866-022-02688-7.

## Introduction

Phosphorous (P) is the second important key plant nutrient which limits plant growth. Phosphorous has no atmospheric reservoir that can be made biologically available. The majority of phosphorous in the soil exists in fixed and/or insoluble forms and hence, unavailable to plant nutrition in soil. P forms insoluble complexes with Al, Fe, and Mn in acidic soils, while in neutral and alkaline soils it reacts strongly with Ca [1]. The existence of soil inhabiting natural phosphate solubilizing microorganisms has been recognized since 1903 [2]. These microorganisms convert inorganic forms of fixed/insoluble Al, Fe, Mn and Ca phosphates to plant available forms through various mechanisms, mainly by producing organic acids that chelate cationic partners of P ions and release PO4−3 directly into soil solution [1]. Phosphate solubilizing microorganisms, such as Bacillus, are the most eco-friendly and inexpensive options for enhancing P availability for plants, once they are capable of transforming insoluble P into soluble (plant accessible) forms and are regarded as plant growth-promoting microorganisms [3].

For several decades, rhizobia have been described as the only exclusive inhabitants of legume nodules [4]. However, several other bacterial taxa which are not typically rhizobia are frequently found within nodules alongside symbiotic rhizobia and are suggested to affect the growth and fitness of the host plant [5]. The other studies also exhibited the presence of diversified bacteria inhabiting in the root nodules of diverse legumes and playing assistance role to the nodulation process and plant growth activities. Not only the rhizospheric and root nodule inducing bacteria but also the passenger endophytic bacteria that are known to be involved in plant growth-promotion and are important in plant development in stressful environmental conditions [6]. These passenger endophytes coexist with Alpha- or Beta-rhizobia in the legume nodules [7]. They are called root nodule bacteria [8]. These days, several names have been proposed by different reports like non-rhizobia endophytes [9], nodule endophytes [10], nodule associated bacteria [11, 12] and most recently Preyanga et al.[13] proposed passenger endophyte bacteria. Martinez-Hidalgo and Hirsch [4] have revealed that rhizobia and the passenger endophytes act together as a community within the root nodules to facilitate plant health and survival, particularly under conditions of environmental stress.

Recently Tapia-García et al. [14] have reported several genera of root nodule associated bacteria with plant growth promoting activities from legume nodules, including *Achromobacter, Acinetobacter, Bacillus, Brevibacillus, Brevibacterium, Dyella, Enterobacter, Herbaspirillum, Kosakonia, Labrys, Microbacterium, Moraxella, Paraburkholderia, Pseudomonas, Stenotrophomonas;* and *Aeromonas, Marinococcus Pseudarthrobacter* and *Pseudoxanthomonas*. Similarly, Soares et al. [15] have isolated several diversified wild Lotus parviflorus root nodule associated bacteria having plant growth promoting traits which comprised of alpha- (Rhizobium/Agrobacterium), beta- (Massilia) and gamma-proteobacteria (*Pseudomonas, Lysobacter, Luteibacter, Stenotrophomonas* and *Rahnella*), as well as some bacteroidetes from genera *Sphingobacterium* and *Mucilaginibacter*. Pang et al. [16] have also reported diverse types of root nodule associated bacteria with plant growth promoting characteristics from the root nodules of diverse legume plants in china. Their report consisted of Bacillales, Rhizobiales, Pseudomonadales, Burkholderiales, Paenibacillales, Enterobacteriales, Actinomycetales, Sphingomonadales, Xanthomonadales, Chitinophagales, Brevibacillales, Staphylococcales, or Mycobacteriales. Most recently Youseif et al.[12] have reported 34 nodule associated/passenger bacteria from the root nodules of faba bean in Egypt. They have revealed that these bacteria are members of Enterobacteriaceae belonging to the genera *Klebsiella*, *Enterobacter *and *Raoultella*. These researchers also found that most of these passenger endophytes possessed plant growth promoting traits and further reported that the co-inoculation of two of these passenger endophytes with rhizobia significantly increased paba bean nodulation, growth and nitrogen uptake compared to single inoculated or un-inoculated treatments. Furthermore, Knežević et al. [17] have reported 44 non-rhizobial isolates from the root nodules of nodules of *Medicago sativa* L. and *Lotus corniculatus* L. and revealed that the majority exhibited indole-3-acetic acid (IAA) production; 29 produced siderophores, few isolates performed phosphate solubilization and/or produced lytic enzymes, while 30% of isolates showed notable antifungal activity. The most promising strains were identified as members of *Bacillus, Pseudomonas* and *Serratia* genera, based on 16S rRNA sequence analysis in their study.

The passenger endophytes obtained from diversified legume plants and agro ecological zones across the globe have exhibited several plant growth promoting traits like increased yield, reduced pathogen infection, improved abiotic and biotic stress tolerance [18], production of plant growth regulators [19], production of osmoprotectants and exopolysaccharides [20], production of antimicrobial metabolites that can function as biocontrol agents [21], improved biological nitrogen fixation [22], enhanced nutrient availability to the host plants [23], modulating plant growth under wide range of stressful environmental conditions such as drought and salt stress [24, 25], production of IAA, ACC deaminase activity, siderophore production and phosphate solubilization [26]. For example, Sturz et al. [27] reported 114 bacteria from the root nodules of Pigeon pea, of which 40% were passenger endophytes. Moreover, Palaniappan et al. [26] have reported out of 39 Lepspedeza sp. root nodule endophytes 24 (61.5%) were phosphate solubilizers. Recently, Tapia-García et al.[14] have studies 83 root nodule associate bacteria of which 32.5% were phosphate solubilizers, while 56.6% were found to be IAA producers.

These days, there has been increasing interest to characterize and bioprospect root nodule endophytes for plant growth promoting traits from diverse legume plants in different parts of the globe [3, 12–14, 16, 17]. These studies are aimed at obtaining potential endophytic inocula and to elucidate functional relationships between endophytes and host plants.

*Erythrina brucei* is an endemic woody leguminous tree widely distributed in different land use types in Ethiopia [28]. The contributions of *E. brucei *to small holder farmers in intercropping and traditional agro-forestry practices in different parts of Ethiopia are well established [29–32]. In addition, the biomass of this particular woody legume has been used as mulching, cover crop and green manure material by small holder farmers to improve soil fertility. The plant has also very attractive agro-forestry attributes such as rapid establishment from seeds and cuttings, possession of spreading canopy, high rate of litter production, very soft woody nature and very fast litter decomposition and mineralization [33] which make this plant best candidate to use as soil fertility improving material due to its symbiotic association with rhizobia for biological nitrogen fixation.

Amsalu et al. [34] have reported rhizobia and passenger endophytic bacteria such as *Bradyrhizobium* spp., *Mesorhizobium* spp., *Rhizobium* spp., *Enterobacter *spp., *Agrobacterium* spp. and *Rahnella* species from the root nodules of E. brucei. Most recently, Berza et al. [29] have reported several symbiotic and passenger endophytic bacteria from the root nodules of E. brucei. These included *Bradyrhizobium* spp., *Enterobacter* spp., *Bacillus* spp., *Paenibacillus* spp., *Staphylococcus* spp. and *Stenotrophomonas* species.

Despite its very important roles in the traditional agro-forestry systems/practices in the southern and southwestern Ethiopia, the root nodules of *E. brucei *are not well explored with respect to isolating and characterizing phytobeneficial microorganism in a perspective of enhancing growth, development and productivity of this particular host plant. Hence, there is a need for more information regarding plant growth promoting traits of root nodule inducing bacteria and endophytic bacteria associated with *E. brucei* to enhance its growth and productivity. Moreover; since the host plant is endemic to Ethiopia, there is very limited information with regard to the roles of endophytic bacteria in the growth, nodulation, development and productivity of *E. brucei*. This study, therefore, aimed to isolate, screen and evaluate inorganic phosphate solubilizing efficiency and other multiple plant growth promoting traits of root nodule passenger endophytic bacteria of *E. brucei*.

## Materials and methods

### Description of the soil sampling locations, nodule induction and bacteria isolation

*Erythrina brucei *rhizosphere soil samples were obtained from 15 different geographic locations in the southern, central and northern Ethiopia. The host plant has been grown for several purposes like shade tree, low cost nutrient source in farmlands, bio-fence/live fence/live land boundary fence and in forests in these sampling locations. The locations of soil sampling points, their climatic conditions and soil types are presented in Table 1. The root nodules were obtained by plant infection method. In brief, In brief, plastic pot of four kg holding capacity were surface sterilized with 70% ethyl alcohol and filled with 3 kg field soil obtained from sampling locations and placed in a greenhouse to trap E. brucei root nodule bacteria in fresh and intact nodules. The seeds of *E. brucei* were collected under *E. brucei* trees from Addis Ababa University, College of Natural Sciences and shade dried and surface sterilized by immersing in 70% ethyl alcohol for 2 min, followed by immersing into 3% sodium hypochlorite solution for 8 min. The surface sterilized seeds were successively washed up to five times with distilled sterile water and then immersed in distilled sterile water and left overnight at room temperature to remove the anti-nutritional factors. Seeds were washed again with distilled sterile water, allowed to germinate on 1% water agar (w/v), and incubated to germination for 7 days at 28 °C. The seedlings were transplanted into plastic pot. The seedlings were watered regularly three times a week either in the morning at 12 AM or in the evening 12 PM for three months. This trapping experiment was conducted in triplicates under ambient light and temperature conditions (about 22–24 °C day/10–14 °C night). The E. brucei plants were uprooted after 90 days of growth and root nodules were harvested. The root nodules bacteria isolation was carried out according to methods described in Berza et al. [29].

**Table 1:** Soil sampling location with their climatic conditions and soil types

Sampling locations	GPS Coordinates of sampling locations		Altitude (m.a.s.l)	Regions	Soil type	Mean annual temperature ( ℃)	Mean annual precipitation (mm)	Land use type
	Latitude	Longitude						
Hossana	07^0^,32’,492’’	037^0^,50’,524’’	2354	SNNPR	Humic Nitisol	19.23	759.38	Farmland
Teza Agara	07^0^,16’,435’’	037^0^,55’,123’’	2349	SNNPR	Humic Nitisol	19.23	759.38	Shade Trees
Bodit	06^0^,57’,429’’	037^0^,51’,341’’	2020	SNNPR	Eutric Vertisol	20.3	1117.97	Farmland
Humbo	06^0^,43’,021’’	037^0^,46’,548’’	1637	SNNPR	Humic Alisol	21.1	1455.47	Farmland
Sodo Town	06^0^,51’,178’’	037^0^,45’,462’’	2101	SNNPR	Humic Nitisol	20.68	1165.43	Shade Trees
Dhakalo	06^0^,12’,915’’	037^0^,19’,618’’	2598	SNNPR	Humic Alisol	21.4	1513.48	Farmland
Gidole	05^0^,38’,464’’	037^0^,21’,642’’	2158	SNNPR	Chromic Luvisol	22.49	912.3	Forest
Debre Markos	10^0^,19’,686’’	037^0^,44’,016’’	2387	Amhara	Humic Nitisol	17.71	1539.84	Shade Trees
AAU	09^0^,02’,082’’	038^0^,045’,964’’	2467	Addis Ababa	Humic Nitisol	16.51	912.3	Shade Trees
Adiskdam	10^0^,33’,027’’	037^0^,74’,502’’	2424	Amhara	Eutric Vertisol	17.32	938.67	Land boundary fence
Injibara	10^0^,97’,589’’	036^0^,92’,776’’	2561	Amhara	Haplic Luvisol	19.51	1492.38	Land boundary fence
Tilil	10^0^,86’,789’’	037^0^,00’,420’’	2453	Amhara	Chromic Luvisol	19.86	1703.32	Land boundary fence
Burie	10^0^,71’,557’’	037^0^,06’,920’’	2122	Amhara	Humic Nitisol	17.68	2093.55	Forest
Rebu Gebeya	10^0^,71’,266’’	037^0^,06’,680’’	2087	Amhara	Humic Nitisol	20.51	1824.61	Forest
Enrata	10^0^,42’,846’’	037^0^,72’,838’’	2500	Amhara	Eutric Vertisol	17.32	938.37	Shade Tree

### Screening for inorganic phosphate solubilization

The root nodule endophytes were screened for their ability to solubilize sparingly soluble inorganic phosphate sources namely tri-calcium phosphate (TCP), ferric phosphate, (Fe–P) and aluminium phosphate (Al-P) according to the methods described Berza et al. [29].

### Production of Iodole acetic acid (IAA)

**Fig. 1:**
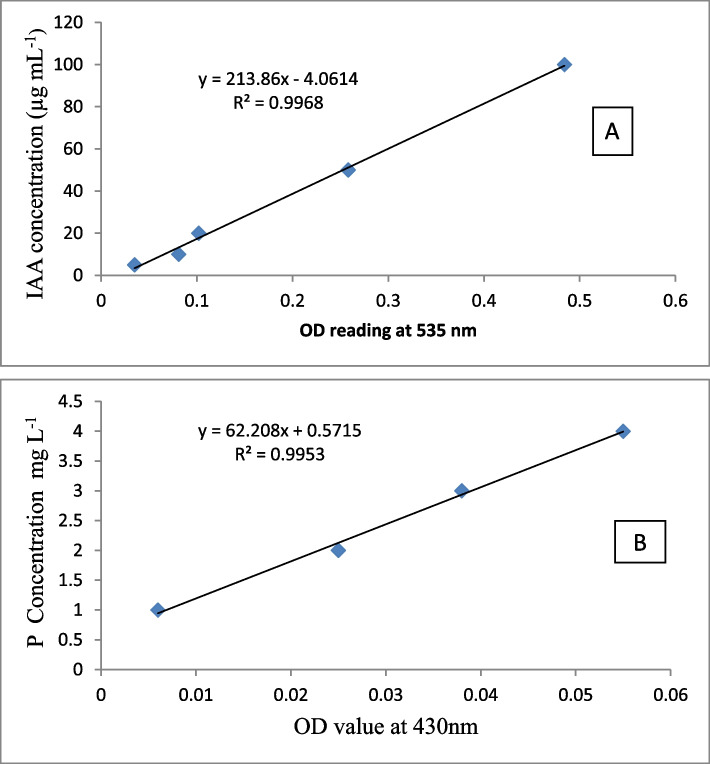
Standard curves for quantification of IAA (A) and solubilized inorganic phosphate (B)

Indole acetic acid (IAA) production by the endophytes was estimated by inoculating 1 mL of 72 h YEM broth composed of g/L; [yeast extract, 0.5; D-mannitol, 10; K2HPO4, 0.5; MgSO4.7H2O, 0.2; NaCl, 0.1] bacterial culture into Luria Bertani (LB) composed of g/L; [tryptone, 10; yeast extract, 5; NaCl, 2.5] supplemented with L-tryptophan (0.1 g/L) as described in Berza et al. [29]. The colorimetric quantification of IAA produced by the selected endophytes was carried out by measuring the absorbance of the resulting solution at 535 nm using spectrophotometer (Jenway, 6405 Uv/vis spectrophotometer, England). The concentration of IAA in the culture supernatant was quantified using a standard curve prepared using various concentrations of analytical grade IAA (Fig.  1A).

### Quantification of phosphate solubilizing potential of the isolates using liquid media

Based on the inorganic phosphate solubilization indices and IAA production potential, nine isolates were selected. Inorganic phosphate solubilizing potential of these selected isolates was determined using National Botanical Research Institute's phosphate (NBRIP) growth liquid medium [35]. In Fe-P and Al-Psolubilization studies, the quantity of TCP was substituted individually by Fe-P or Al-P [33]. Each isolate was grown to 72 h in YEM broth from which 10 µl (108 cell mL-1) suspensions was inoculated into 20 mL NBRIP liquid medium in fifty mL capacity flasks. The NBRIP medium was composed of (g/L) ; [glucose, 10 ; Ca3(PO4)2, 5 ; MgCl2.6H2O, 5 ; MgSO4.7H2O, 0.25; KCl, 0.2 and (NH4)2SO4, 0.1]. The pH of each medium was adjusted to neutral before the experiment. The un-inoculated controls were included as control. All flasks were incubated at 28 ◦C with gentle shaking at 120 rpm for six consecutive days. A 10 mL culture was removed and centrifuged at 12,500 rpm for 10 minutes and the supernatant was used for determining pH and the amount of phosphate released in the medium. The released phosphate was determined using colorimetric method. In brief, the supernatant obtained by centrifugation was filtered through a 0.45 µM millipore filter and 0.1 ml of the supernatant was mixed with 0.25 ml of Barton’s reagent and the volume was made to 5 ml with distilled water. After 10 min, the intensity of yellow color was read using spectrophotometer (Jenway, 6405, UV–VIS Spectrophotometer, England) at 430 nm and the amount of P-solubilized was extrapolated from the standard curve (Fig. 1B ). The standard curve was prepared using various concentrations of analytical grade KH_2_PO_4_ (Fig.1B).

The quantification was carried out by subtracting the phosphate released in un-inoculated controls which could be due to autoclaving from the bacteria inoculated cultures. The data used in this research are means of three independent experiments that were conducted in similar experimental conditions.

### Hydrogen cyanide (HCN) and Ammonia (NH_3_) production by the isolates

Production of HCN was measured qualitatively according to methods described in [33]. Production of HCN was indicated by the change in color of the filter paper strip from yellow to brown to red. The intensity of the color change was recorded qualitatively as (++ - red, + -brown and –yellow for non-production). The production of NH_3_ was determined according to the method described in [36]. Formation of yellow to brown precipitate was indication of the presence of NH_3_ in the culture by the bacterial strains.

### Synthesis of hydrolytic enzymes

Chitinase production was evaluated according to the methods described by Saima et al. [37]. The bacteria colonies with clear halo zone around creamish background were considered as chitinase-producing bacteria. Protease production study was carried out following the procedures described in Dinesh et al. [38]. Plates were observed for clear zone around the colonies after 72 h incubation. Moreover, lipase production was done according to the methods described in Smibert et al. [39]. After 7 days of incubation, production of opaque zone around colony was indicated as lipolytic activity of the bacterial strains.

### Genomic DNA extraction and identification of bacterial isolates

The genomic DNA of the selected bacteria isolates was extracted individually according to the methods described in Berza et al. [29]. The 16S rRNA gene amplification was carried out by using primers described [40].The primers were fD1 (5’-AGTTTGATCCTGGCTCAG-3’) and rP2 (5’-ACGGCTACCTTGTTACGACTT-3’). The amplification was carried out in a 20 µl volume reaction. Sequencing was carried out at Eurofins Genomics (Karnataka, India) using the same primers as used during PCR amplification. The partial 16S rRNA gene sequences of our bacterial isolates were blasted and compared with the nucleotide sequences available at the Genbank database of National Center for Biotechnology Information (NCBI). The partial 16S rRNA gene sequences of our bacterial strains were deposited in the NCBI database under accession numbers between MK370558 to MK370566. The phylogenetic tree was constructed by comparing 16S rRNA sequences of our strains and closest type strains from the NCBI Genbank. The sequence comparison was made by multiple alignments of sequences from this study and those obtained from Genbank. The ClustalW algorithm was used for the sequence alignment, and Neighbor-Joining method was employed for the phylogenetic tree construction. The phylogeny was tested by the bootstrap values of 1,000 replications. The sequence alignments and tree construction were made by using MEGA 7.0 software.

### The effect of bacterial isolates on wheat plant growth

To study the effects of selected endophytic bacteria on the growth and development of wheat, plant experiments were carried out in the greenhouse condition. Based on the phytobeneficial properties particularly, inorganic phosphate solubilization efficiency and IAA production, three isolates; AU4, RG5 and RG6 were selected for the plant experiments. The inorganic phosphate TCP was used as P source. The experiment consisted of five treatments each having four replications. The treatments were; TCP without bacterial inoculation-T1 was considered as negative control and KH_2_PO_4_ without bacterial inoculation-T2 considered as positive control. TCP + AU4-T3; TCP + RG5-T4 and TCP + RG6- T5. Twenty plastic pots of 10.8 cm diameter and 20 cm height were labeled with each treatment and 1.0 kg autoclaved and oven-dried river sand was added per pot. A half (0.5%) TCP per pot sand was added in each of the 16 pots excluding four pots of the positive control treatment which received the same amount of KH_2_PO_4_. The seed surface sterilization was carried out as follows: 80 seeds of wheat variety (Ogolcho) were soaked in 100 ml of 10% bleach solution for 15 min, and six times successively washed using distilled sterile water for 10 min. The seeds were carefully transferred to 1% (w/v) water-agar plates using sterile forceps and incubated at 28 ◦C for 3 days to allow germination. The endophytic bacteria isolates were individually inoculated in 50 ml nutrient broth and incubated in shaking incubator at 120 rpm, at 28 ◦C for 24 h. After 24 h, the optical density of each strain was adjusted at 1.0 at the wavelength of 600 nm. The bacterial cells were harvested from 40 ml culture at 5,000 rpm for 20 min and resuspended in 0.85% saline (P source excluded), containing 108 cells/ml. Each pot was fertilized with forty milliliter sterile full strength (P source excluded) nitrogen containing Hoagland’s solution [41] every seven days. The Hoagland’s solution was prepared by using distilled sterile water. The 3-days old seedlings were transferred in to each pot using sterilized forceps and 1 ml bacterial was inoculated to each respective pot excluding negative and positive controls. The pots were arranged in a completely randomized design. The mean temperature was 25 ± 2◦C and 12 h day and 12 h dark. Plants were watered at alternate days using distilled sterile water for 30 days. Plants were uprooted after 30 days of growth and roots were washed using tap water and separated from the shoots. The following biometric data: shoot length and dry weights, root length and dry weights and the number of tillers produced per seedling were recorded for all samples. This experiment was conducted in triplicates with four replications. This evaluation experiment was carried out at DebreMarkos University, Department of Biology greenhouse from February to April, 2022.

### Statistical data analysis

One way analysis of variance was employed to test significant differences in inorganic phosphate solubilizing efficiency, phosphate solubilization indices, plant biometric data and IAA production potential among selected bacteria species using SAS version 9.4. Duncan’s multiple range test was conducted to test for mean separation (*p* < 0.05).

## Results

### Isolation and screening the isolates for phosphate solubization potential

In this study, a total of 304 bacterial endophytes were recovered from the root nodules of E. brucei. These endophytic bacteria were screened for the solubilization potential of sparingly soluble inorganic phosphates. The isolates exhibited variations in solubilizing different phosphate sources; TCP, Al-P and Fe–P supplemented in Pikovskaya Agar (PA) medium. Total of 119 (39.14%) isolates exhibited clearly visible halos zones around colonies on PA medium supplemented with TCP (supplementary Fig. 1). The phosphate solubilization indices formed by the isolates varied between 0.5 and 6.0 (supplementary Table S1). Nine (9) (7.6%) isolates produced PSI greater than or equals to 4, 67 (56.3%) isolates produced PSI between 2.0 and 3.9, while 43(36.1) isolates produced PSI less than 2.0. All TCP solubilizing bacteria exhibited growth on PA medium supplemented with Al-P or Fe–P; however, none of these bacteria exhibited visible halos on the PA media supplemented with Al-P or Fe–P.

### Screening inorganic phosphate solubilizing endophytes for IAA production

The 119 root nodule bacterial endophytes that exhibited inorganic phosphate solubilization potential were also screened for IAA production potential using Luria Bertani (LB) medium supplemented with L-tryptophan. Forty eight (48), (40.3%) of the isolates showed IAA production potential through formation of pink coloration after incubation of the culture supernatant mixed with Salkowski reagent for 30 min in a dark place. Based on the phosphate solubilization and IAA production capabilities, nine potential isolates were selected for further quantification of solubilized phosphate and IAA production. These bacterial isolates included; AU4, BU2, DM17, EN5, EN6, GH6, RG5, RG6 and TL3.

### Phylogenetic position of potential isolates using 16S rRNA gene sequence analysis

The partial 16S rRNA gene analysis has identified and placed these bacteria to their phylogenetic positions. The 16S rRNA gene sequence similarity of the bacterial isolates and the reference strains is presented in Table 2.

**Table 2:** Identity of the isolates based on 16S rRNA gene sequences analysis

Isolate	Isolate query length (bp)	Isolate identified as	Accession Number	Best match ID(NCBI ref)	Query Coverage (%)
AU4	1422	*Acinetobacter soli*	MK370560	APPU01000012	99
BU2	1367	*Gluconobacter cerinus*	MK370563	BEWM01000030	100
DM17	1427	*Bacillus thuringiensis*	MK370566	ACNF01000156	100
EN5	1367	*Gluconobacter cerinus*	MK370564	BEWM01000030	100
EN6	1367	*Gluconobacter cerinus*	MK370565	BEWM01000030	100
GH6	1414	*Achromobacter xylosoxidans*	MK370558	CP006958	99
RG5	1422	*Acinetobacter soli*	MK370559	APPU01000012	99
RG6	1422	*Acinetobacter soli*	MK370561	APPU01000012	99
TL3	1367	*Gluconobacter cerinus*	MK370562	BEWM01000030	100

**Fig. 2:**
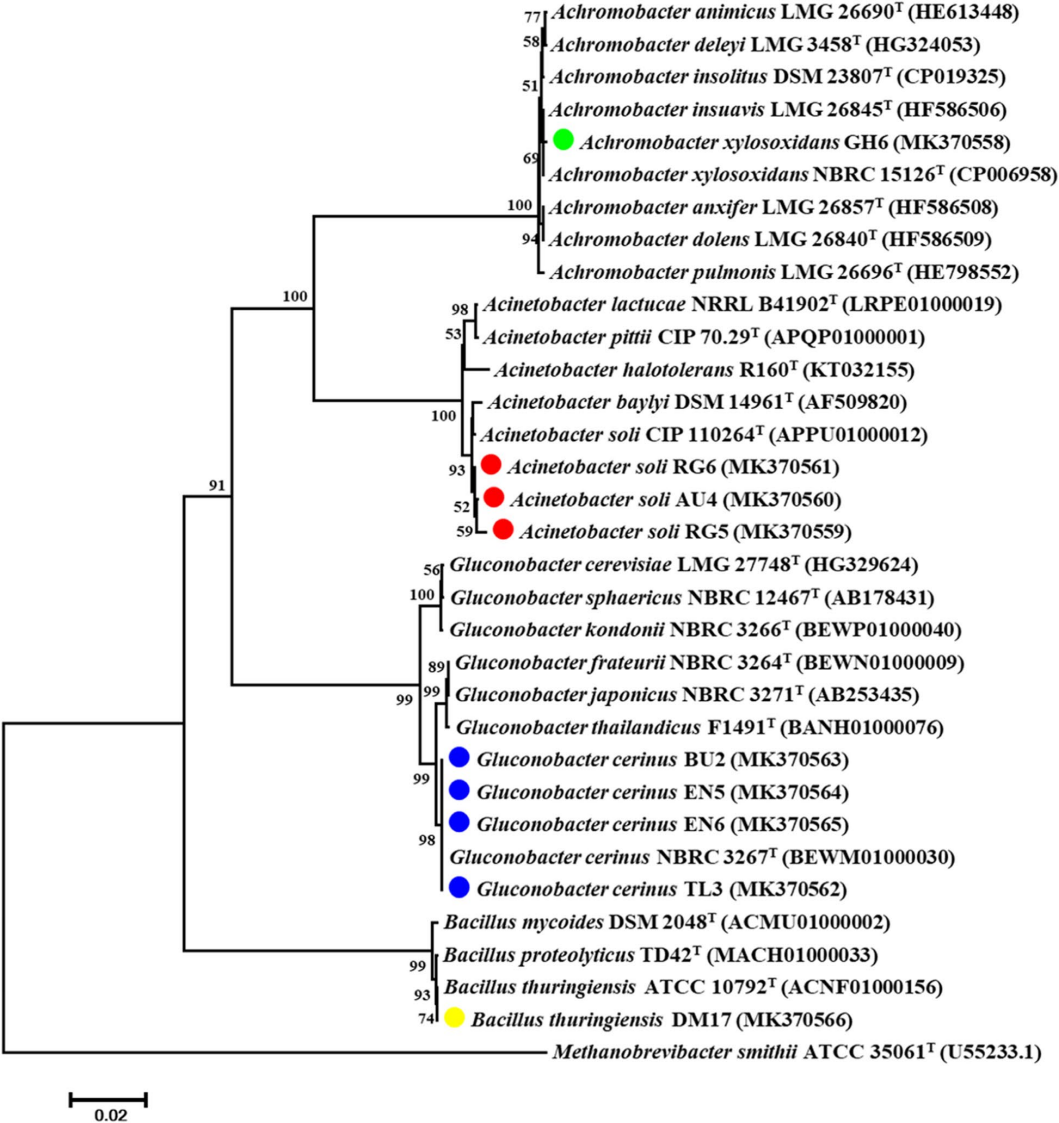
The phylogenetic relationship between the inorganic phosphate solubilizing strains and reference strains from genbank based on 16S rRNA gene sequences. The genbank accession numbers of the strains are presented in the parentheses

Based on a sequence identity of 97% or greater [42] our isolates were affiliated to Firmicutes (1) and Proteobacteria (8). These eight proteobacteria were distributed into the beta (1), gamma (3) and alpha (4) sub-divisions of the Proteobacteria. Our isolates are grouped into four genera; *Achromobacter*, *Acinetobacter*, *Gluconobacter* and *Bacillus *(Fig. 2). All these genera are the first reports from root nodules of* E. brucei*.

Isolate GH6 is clustered to genus *Achromobacter*. This isolate was obtained from Gidole (forest land use type), south Ethiopia (Fig. 2). Likewise, isolates AU4, RG5 and RG6 are grouped to genus *Acinetobacter*. However, they were obtained from different sampling locations. AU4 was recovered from root nodules in Addis Ababa (shade land use type), central Ethiopia, whereas isolates RG5 and RG6 were isolated from Rebu gebeya (forest land use type), north Ethiopia. The other four isolates BU2, EN5, EN6 and TL3 are grouped to genus *Gluconobacter* (Fig. 2). These isolates were recovered from Burie (forest), Enrata (shade tree), and Tilil (bio-fence/live fence), respectively and all from north Ethiopia. Moreover, isolate DM17 is clustered to genus *Bacillus*. This isolate was also recovered from Debre Markos (shade tree), north Ethiopia.

### Evaluation of inorganic phosphate solubilization efficiency using liquid media

The inorganic phosphate solubilizing efficiency of the selected isolates was quantified in NBRIP liquid medium individually supplemented with TCP, Al-P or Fe–P. All these selected bacterial isolates exhibited inherent phosphate solubilization capacity to the all inorganic phosphate sources. The phosphate solubilization potential of the isolates was exhibited by increment in the amount of solubilized phosphate released in to the liquid NBRIP medium along the incubation periods (Table 3). The net solubilized phosphate is reported by subtracting the quantity of phosphate released in the control medium which could be probably released due to autoclaving. In this case, 24.21 mg L^−1^, 7.04 mg L^−1^ and 4.30 mg L^−1^ phosphorous was released from TCP, Al-P and Fe–P, respectively due to probably autoclaving.

**Table 3:** Inorganic phosphate solubilization efficiency of E*. brucei* root nodule endophytesin liquid NBRIP medium during six days of incubation

Isolate	Taxonomic group	PA (solid) SI	Tricalcium phosphate[Ca_3_(PO4)_2_]				Aluminum phosphate [ AlPO_4_]				Iron phosphate [FePO_4_]			
			Net P released Day 3 (mgL^−1^)	Net P increased at day 3 by	Net P released Day 6 (mgL^−1^)	Net P increased at day 6 by	Net P released Day 3 (mgL^−1^)	Net P increased at day 3 by	Net P released Day 6 (mgL^−1^)	Net P increased at day 3 by	Net P released Day 3 (mgL^−1^)	Net P increased at day 3 by	Net P released Day 6 (mgL^−1^)	Net P increased at day 3 by
AU4	*A. soli*	6.00a	108.96a	4.50	120.36a	4.97	87.33a	12.40	99.66b	14.15	84.04a	19.54	93.22b	21.67
BU2	*G. cerinus*	4.12f	106.76 cd	4.41	111.17c	4.59	84.23b	11.96	98.35b	13.97	80.95b	18.83	81.20e	18.88
DM17	*B. thuringiensis*	5.00c	104.62ed	4.32	109.48d	4.52	77.78c	11.05	91.91d	13.05	78.50d	18.26	89.34c	20.77
EN5	*G. cerinus*	4.20f	102.87f	4.25	106.73f	4.40	77.32c	10.98	89.57e	12.72	82.05b	19.08	95.14ba	22.12
EN6	*G. cerinus*	4.44d	104.71ed	4.33	107.91e	4.45	77.10c	10.95	95.12c	13.51	81.38b	18.93	82.83ed	19.26
GH6	*A. xylosoxidans*	5.40b	103.73ef	4.28	106.03f	4.37	64.18e	9.12	91.85d	13.04	81.10b	18.86	88.68c	20.62
RG5	*A. soli*	4.00 g	105.60 cd	4.36	109.97d	4.54	27.12f	3.85	45.12f	6.40	40.46e	9.41	48.85f	11.36
RG6	*A. soli*	4.33ed	107.48b	4.44	112.82b	4.66	84.12b	11.95	102.14a	14.50	79.68c	18.53	96.07a	22.43
TL3	*G. cerinus*	4.30df	100.82 g	4.16	103.57 g	4.27	74.97d	10.65	94.24c	13.38	81.71b	19.00	83.56d	19.43

*PA* Pikovskaya agar, *SI* Solubilization index, *NBRIP* National Botanical Research Institute Phosphate medium. Values are expressed as means of three independent experiments. Means sharing the same letters in the same column do not differ significantly at *p* ≤ 0.05 by ANOVA Duncan test

### Evaluation of phosphate solubilizing efficiency of isolates using TCP

In this study, we recorded significant (*p* < 0.05) difference among the amount of net solubilized phosphate released by the isolates (Table 3). The highest amount of solubilized that correspond to 108.96 mg L^−1^and 107.48 mg L^−^1 were recorded by strains AU4 and RG6 (Acinetobacter soli), respectively on the third day. We recorded increments in the amount of solubilized phosphates released by each isolate at the sixth day of sampling. Strains AU4 and RG6 (*Acinetobacter soli*) solubilized the highest amount, 120.36 mg L^−1^ and 112.82 mg L^−1^, respectively. Almost all the isolates solubilized quantitatively a greater amount of phosphorous from TCP at sampling day 6 compared to day 3. The isolate AU4 (*Acinetobacter soli*) solubilized significantly (p < 0.05) higher phosphate throughout the sampling periods compared to the other isolates.

### Evaluation of phosphate solubilizing efficiency using Al-P

All the selected isolates exhibited inherent Al-P solubilization potential in the liquid medium. We recorded significant (*p* < 0.05) variations in Al-P solubilizing abilities among isolates. None of these isolates formed clear halos around colonies in the solid PA medium supplemented with Al-P. However, all of these isolates grew well in PA medium supplemented with aluminum phosphate as sole P source. The highest amount of solubilized phosphate, 87.33 mg L^−1^ was recorded by isolate AU4 (*Acinetobacter soli*) followed by isolate BU2 (*Gluconobacter cerinus*) which solubilized 84.23 mg L^−1^ (Table 3) on the sampling day 3. Similarly, the highest amount of solubilized phosphate, 102.14 mg L^−1^ was recorded by isolate RG6 (*Acinetobacter soli*) followed by AU4 (*Acinetobacter soli*) which solubilized 99.66 mg L^−1^ (Table 3) at sampling day 6.

### Evaluation of phosphate solubilizing efficiency using Fe–P

Similar to TCP and Al-P solubilization studies, we also evaluated Fe–P solubilizing potential of these isolates using solid PA medium. Like Al-P, none of these isolates formed visible clear halos zones around colonies on solid PA medium supplemented with Fe–P. However, all the isolates grew well on PA medium supplemented with Fe–P. On the other hand, all the evaluated isolates exhibited potential to solubilize Fe–P in the liquid medium and exhibited variations in Fe–P solubilizing ability.

The highest quantity of solubilized phosphate, 84.04 mg L^−1^ was recorded by isolate AU4 (*Acinetobacter soli*) followed by 82.05 mg L^−1^ by isolate EN5 (*Gluconobacter cerinus*) during sampling day 3 (Table 3). Likewise, the highest amount of solubilized phosphates were recorded by isolates RG6 (*Acinetobacter soli*) and EN5 (*Gluconobacter cerinus*) which correspond to 96.07 mg L^−1^ and 95.14 mg L^−1^ respectively at sampling day 6. All these evaluated isolates exhibited progressive increment in the amount solubilized phosphate released across the sampling days (Table 3). Similar medium pH reduction trends were recorded in Fe–P supplemented medium like other phosphate sources.

## Quantification of IAA producing efficiency of the selected isolates

The isolates were further evaluated for their IAA quantification in LB medium supplemented with tryptophan. Each isolate produced significantly (*p* < 0.05) different amount of IAA. The quantity of IAA produced by each bacterium is presented in Table 4. The highest amount of IAA 0.313 mg mL^−1^ was produced by isolate DM17 (*Bacillus thuringiensis*) followed by EN6 (*Gluconobacter cerinus*) which exhibited about 0.266 mg mL. The smallest amount of IAA, 0.075 mg mL^−1^ was recorded by BU2 (*Gluconobacter cerinus*).

**Table 4:** Multiple Plant growth promoting traits of selected inorganic phosphate solubilizing endophytes recovered from the root nodules of *E.brucei*

Isolate	Taxonomic position of the isolates	IAA(µg/mL)	HCN	NH_3_	chitinase	protease	lipase
AU4	*Acinetobacter soli*	171.65c	+ +	+	-	+	+
BU2	*Gluconobacter cerinus*	75.07i	+ +	+	+	-	+
DM17	*Bacillus thuringiensis*	313.61a	+ +	+	+	-	+
EN5	*Gluconobacter cerinus*	112.57f	+ +	+	+	-	+
EN6	*Gluconobacter cerinus*	266.29b	+ +	+	-	+	+
GH6	*Achromobacter xylosoxidans*	147.28d	-	+	+	+	+
RG5	*Acinetobacter soli*	120.65e	-	+	+	+	-
RG6	*Acinetobacter soli*	104.55 h	+ +	+	+ +	+	+
TL3	*Gluconobacter cerinus*	106.37 g	+ +	+	-	+	+

+  + -Red, +—brown/ brown precipitate,—no visible production sign. Values are expressed as mean of three independent experiments. Means with the same letter in the same column are not significantly different at *P* ≤ 0.05 by Duncan test

### Evaluation the selected isolates for other PGP traits

We also evaluate these bacterial isolates for other multiple plant growth promoting traits such as production of HCN, NH3 and hydrolytic enzymes. All the bacterial isolates exhibited HCN and NH3 production except RG5 and RG6 (*Acinetobacter soli*) (Table 4). Moreover, the isolates BU2 (*Gluconobacter cerinus*), DM17 (*Bacillus thuringiensis*) and EN5 (*Gluconobacter cerinus*) have exhibited strong HCN production. Similarly, isolates RG 5 (*Acinetobacter soli*), TL3 (*Gluconobacter cerinus*) and RG6 (*Acinetobacter* soli) showed strong NH3 production potential (Table 4). Moreover, these isolates exhibited inherent potential to the synthesis of fungal pathogen cell wall degrading enzymes such as chitinase, protease and lipase. Each isolate exhibited the production of at least two hydrolytic enzymes; however, almost all of the isolates were weak producers of hydrolytic enzymes (Table 4). The isolate RG6 (*Acinetobacter soli*) was strong producer of chitinase as indicated by halo zone around its colonies on chitinase test. This is the only isolate exhibited the synthesis of all the three hydrolytic enzymes (Table 4).

### The plant experiment

The phytobeneficial endophytic bacteria inoculation improved wheat plant growth under phosphorous deficient sand culture in the greenhouse condition. These growth enhancements are recorded through root and shoot length, root and shoot dry weight and by the number of tillers produced.

In this experiment, we recorded enhanced growth in root and shoot length compared to the negative control plants. The recorded mean root length ranged between 9.56 cm in the negative control plants and 14.37 cm in the TCP + AU4 inoculated plants (Table 5; supplementary Fig. 2). All treatments that consisted of endophytic bacteria inoculation exhibited significant (p < 0.05) difference in root length compared to negative control. Plants inoculated with, AU4, RG6 and RG5 exhibited root length increment by 50.3%, 45% and 35.9%, respectively compared to the negative control (Table 5). With regard to shoot length, the highest mean shoot length was recorded by the positive control plants (29.81 cm) followed by treatment consisted of RG6 + TCP (28.56 cm) (Table 5). Inoculation of wheat plant with AU4, RG6 and RG5 increased shoot length by 11%, 17.4% and 14.6%, respectively compared to the negative control plants.

**Table 5:** Wheat growth parameters after 30 days of *E. brucei* root nodule endophytic bacteria inoculation

Treatment	Root length (cm)	Shoot length (cm)	Number of tillers(number)
TCP	9.56 ± 1.81^b^	24.31 ± 4.31^b^	00. ± 00^b^
KH_2_PO_4_	10.62 ± 3.05^b^	29.81 ± 2.26^a^	0.75 ± 0.68^a^
TCP + AU4	14.37 ± 3.73^a^	27.00 ± 5.18^ba^	0.75 ± 0.70^a^
TCP + RG5	13.00 ± 2.65^a^	27.87 ± 5.68^ba^	0.25 ± 0.44^ba^
TCP + RG6	13.87 ± 2.91^a^	28.56 ± 4.70^ba^	0.37 ± 0.50^ba^

**Fig. 3:**
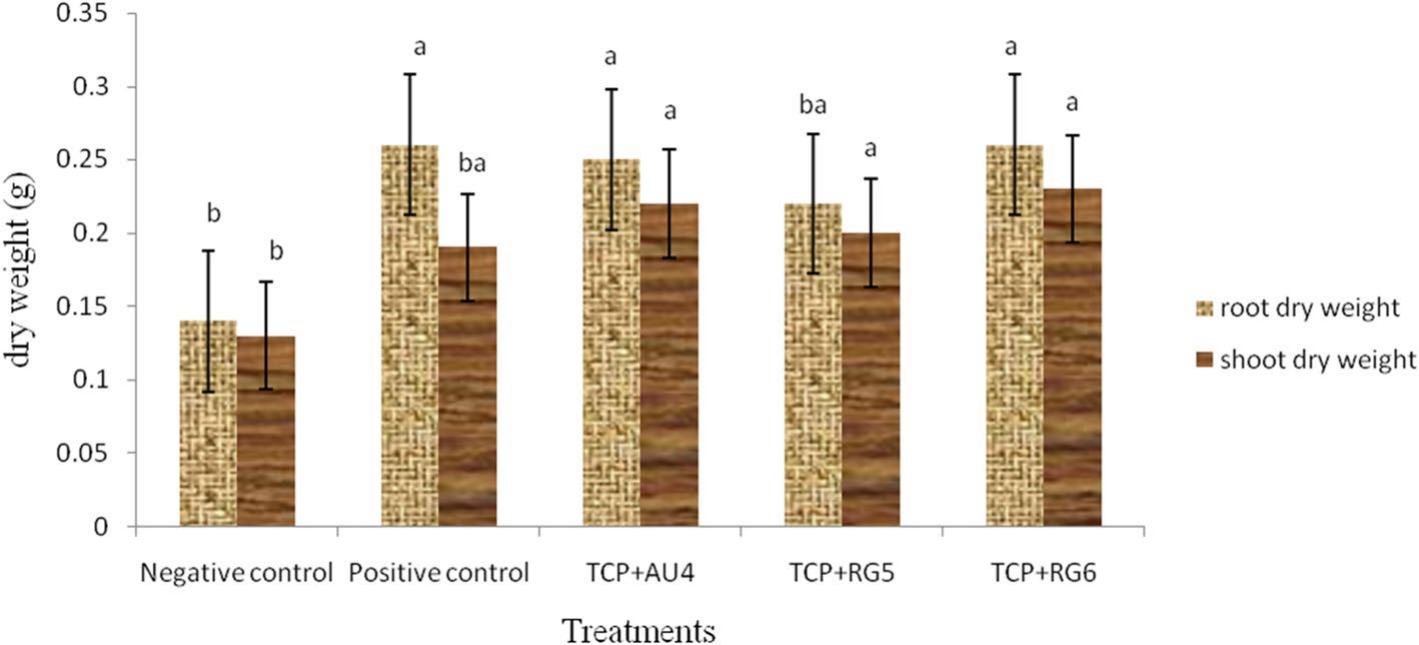
Shoot and root dry weight of wheat plant due to inoculation with *E. brucei* root nodule endophytes bacteria after 30 days of growth under greenhouse condition

We observed a significant (*p* < 0.05) difference in root and shoot dry weight among treatments (Fig. 3). The highest root dry weight is recorded by the positive control plants and inoculation treatment consisted of TCP + RG6 (0.26 g each). However, the highest shoot dry weight is exhibited by the treatments consisted of TCP + RG6 followed by TCP + AU4, 0.23 g and 0, 22 g, respectively. Even though there is no significant difference in the shoot dry weight among treatments consisted of endophytic bacteria, inoculation exhibited significant difference when compared to the positive control (Fig. 3). AU4, RG6 and RG5 inoculations increased root dry weight by 78.5%, 87.7% and 57.1%, respectively and shoot dry weight by 69.2%, 76.9% and 53.8%, respectively compared to the negative control. Moreover, AU4, RG6 and RG5 inoculated wheat plants exhibited 22.6%, 21.3%, 5.3% increment in shoot fresh weight and 15.7%, 21% and 5.2% in shoot dry weight, respectively compared to the positive control plants.

In this experiment, we observed significant (*p* < 0.05) differences among treatments with respect to tiller production in wheat plants (Table 5). No tiller production was recorded in the negative control wheat plants. All treatments produced tillers except the negative control. The highest mean number of tillers were recorded in the positive control plants and treatments consisted of TCP + AU4 (0.75 each) (Table 5).

## Discussion

In the present study, we recorded 39% inorganic phosphate solubilizing endophytes from the root nodules of *E. brucei*. Youseif et al. [12] have evaluated 34 endophytic strains recovered from faba bean root nodules and 27 strains exhibited multiple plant growth promoting traits. They have also reported that 100% strains were able to solubilize inorganic phosphorus within the range of 10–136 μg/mL Similarly Palaniappan et al. [26] have reported 61.5% phosphate solubilizing Lespedeza sp. root nodule endophytes. More recently, Tapia-Garcia et al. [14] also reported 32.5% phosphate solubilizing root nodule endophyte that supports our findings. In our study, all these bacteria exhibited clearly visible halo zones on PA plates containing TCP. The existence of soil microbes endowed with inorganic phosphate solubilizing capabilities in alkaline and/or acidic soils is very important asset and it has to be also noted that these root nodule associated bacteria exist as saprophytes in the soil in the absence of their host plants. Therefore, these endophytic microbes can solve P nutrient deficit by solubilizing and releasing P locked in Al-P, Fe–P and Mn-P in acidic soils and Ca-P in alkaline soil [1]. The 43% of Ethiopia soils are acidic [43], hence in these acidic soils the inorganic phosphates are found adsorbed into sparingly soluble precipitates of Fe–P and Al-P [44]. Therefore, it is imperative to consider acidic soils of Ethiopia, while isolating phosphate solubilizing microbes (rhizospheric and root nodule associated) as part of tropical and subtropical soils.

We included two different synthetic inorganic phosphate compounds (Al-P and Fe–P) in the preliminary screening representing sparingly soluble inorganic P sources which commonly found in soils as sole P sources. All the 119 bacterial isolates showed growth on PA medium supplemented with of Fe–P or Al-P, however, none of these formed visible clear halo zones around colonies. Dinic et al. [45] have reported French bean root nodule bacteria that grew well on plates containing Al-P or Fe–P but did not form visible clear halos around colonies. The growth of bacterial colonies without exhibiting visible halos on plates containing Al-P or Fe–P might be attributed to the solubilization of very small amount of P which could be consumed by the bacterial isolates for their immediate growth [46, 47].

In this study, among these 119 endophytes, 7.6% of phosphate solubilizing bacterial formed PSI values ≥ 4.0, 56.3% formed PSI values 4 > PSI > 2.0, and 36.1% formed PSI values less than 2.0 (Supplementary Table S1). According to Marra et al. [48] these 7.6%, 56.3% and 36.1% of our isolates fall into high, intermediate and low phosphate solubilizers, respectively. The presence phosphate solubilizing bacteria in the root nodule of *E. brucei* has been previously reported by [29, 33, 34]. This preliminary observation revealed the presence of potential inorganic phosphate solubilizing bacterial population in the root nodules of *E. brucei* which can be applied as low cost microbial inputs to enhance the growth, development and productivity of this particular host plant and enrich its biomass with P.

In this study, all the root nodules regardless of the geographic location harbored phosphate solubilizing endophytic bacteria. Variations in the type and distribution of phosphate solubilizing bacteria with soil, climate and cropping history have been well documented [49, 50]. With regard to diversity, the selected phosphate solubilizing bacterial isolates are clustered in to four genera namely: *Achromobacter, Acinetobacter, Bacillus* and *Gluconobacter*. All these genera are the first reports from the root nodules of *E. brucei*. Similar to our findings, recently, Tapia-García et al. [14] have reported several genera root nodule associated bacteria with plant growth promoting activities from legume nodules, including *Achromobacter, Acinetobacter,* and *Bacillus*.

All the evaluated isolates further confirmed the existence o inherent inorganic phosphate solubilization potential by releasing variable amount of solubilized phosphate into NBRIP liquid medium. The phosphate solubilization in liquid media supplemented with TCP was concomitant to medium pH drops and hence phosphate solubilization seems to be due to the medium acidification which could be associated to either proton extrusion or organic acid secretion by bacterial isolates [51, 52]. With regard to the quantity of solubilized phosphate released into NBRIP medium. Chung et al. [40] have reported comparable quantity to our findings. In this study, we did not observe direct correlation between phosphate solubilization indices on the PA plates and the amounts of quantified solubilized phosphate in liquid medium supplemented with TCP. This may imply that the PSI values formed on agar plates do not necessarily guarantee phosphate solubilization efficiency in liquid medium.

We also evaluated the phosphate solubilizing ability of the selected endophytic bacteria isolates in Al-P or Fe–P supplemented NBRIP liquid medium. All our selected isolates have shown mobilization of P from these insoluble inorganic phosphates in liquid media. Even though these bacterial strains did not exhibit clear and visible halos on Al-P or Fe–P on solid medium, they solubilized insoluble inorganic phosphates NBRIP liquid medium. It has been previously demonstrated that many bacterial isolates which did not produce visible halos on agar plates were able to solubilized different types of insoluble phosphates in liquid media [53]. We recorded a higher inherent Al-P solubilization potential by our bacterial isolates compared to [40] who have reported isolates that solubilized P between 3.7 and 13.8 mg L^−1^. As seen in TCP solubilization, we also recorded drops in the medium pH across sampling periods which are concomitant with increased Al-P phosphate solubilization. Several reports have indicated similar medium pH dropping patterns in Al-P supplemented medium [45, 52]. In the case of Fe–P solubilization, our endophytic bacteria isolates exhibited between nine and nineteen times higher Fe–P solubilization at sampling day 3 and between eleven and twenty two times higher at sampling day 6 (Table 3) compared to the controls. The similar medium pH dropping trends were also recorded in Fe–P supplemented NBRIP medium. Unlike the other two phosphate sources, we did not record significant correlation between pH drops and the amount of solubilized phosphate in the case of Fe–P solubilization apart from medium acidification. This may indicate the presence of alternative and different phosphate solubilization mechanisms by these endophytic bacteria. Muleta et al. [53] have suggested that the medium acidification could be due to synthesis of diverse organic acids by the bacterial isolates by consuming the original carbon sources in the medium.

About 40.3% of our phosphate solubilizing isolates produced IAA. In similar manner, Tapia-García et al. [14] have studies 83 root nodule associated bacteria of which 56.6% were found to be IAA producers. In addition, Knežević et al. [17] have reported 44 root nodule endophytic isolates from the root nodules of Medicago sativa L. and Lotus corniculatus L. and the majority exhibited indole-3-acetic acid (IAA) production. With regard to the quantity of synthesized IAA, the quantitified amounts of IAA produced were varied among the bacterial isolates. Amsalu et al. [34] [8] have reported 88% IAA producers from the root nodules of E. brucei. IAA has been implicated in every aspect of plant growth, development and phytopathogen defense responses [54]. Indeed, IAA production is dependent on the presence of enzymatic pathways in the bacteria being studied and concentration of tryptophan supplied in the media [55]. Moreover, the inherent ability of bacteria to produce IAA in the rhizosphere depends on the availability of precursor molecules and uptake of microbial produced IAA by plants [56].The findings in this particular research have a direct practical implication to enhance the growth, development and productivity of E. brucei for improved agro-forestry practices in Ethiopia. Therefore, IAA producer strains from present study can be used in combination with rhizobia and/or the other bacteria endowed with other phytobeneficial traits in the enhancement of the growth and development *E. brucei* and enrichment of its biomass with P for improved agro-forestry practices.

Most of our endophytic bacteria exhibited multiple plant growth promoting traits apart from phosphate solubilization and IAA production. Our isolates are also HCN, NH3 and different hydrolytic enzymes producers. The 77.7% of the tested isolates exhibited HCN production. Youseif et al. [12] have recently isolated 34 endophytic bacteria from faba bean root nodules and reported 100% endophytic bacteria strains were able to produce ammonia. HCN which is volatile secondary metabolite plays significant role in inhibiting growth of plant pathogenic fungi [57]. HCN produced by bacteria is potent inhibitor of cytochrome C oxidase and metaloenzymes and hence affect the respiratory systems of the plant pathogenic fungi [57]. We also recorded hydrolytic enzymes namely; protease, lipase and chitinase production by our bacteria isolates. In this respect, 88.8%, 66.6% and 66.6% of our isolates exhibited lipase, protease and chitinase production, respectively (Table 4). Knežević et al. [17] have evaluated 44 endophytic bacteria isolates from the root nodules of nodules of Medicago sativa L. and Lotus corniculatus L. They have reported few isolates exhibiting lytic enzymes production, while 30% of isolates showed notable antifungal activity. Previously, Amsalu et al. [34] have also reported protease and lipase producing E. brucei root nodule bacterial endophytes. In addition, several reports have indicated the involvement of these enzymes in the fungal cell wall and cell lyses [38, 58–60].

The inoculation of wheat plant with our endophytic bacteria; AU4, RG6 and RG5 significantly (*p* < 0.05) improved plant growth, development and productivity. The inoculation of AU4, RG6 and RG5 increased the root length by 50.3%, 45% and 35.9%, respectively compared to the un-inoculated control. The same inoculation improved root dry weight by 78.5%, 87.7% and 57.1%, respectively compared to the negative control plants. In addition to this, inoculation with AU4, RG6 and RG5 increased shoot length by 11%, 17.4% and14.6% and shoot dry weight by 69.2%, 76.9%and 53.8%, respectively compared to the negative control plants. Such an improved growth, development and productivity in wheat plant could be attributed to the multiple plant growth promoting traits of the inoculated endophytic bacteria strains. The IAA synthesis by our endophytic bacteria probably increased root hair formation, root surface area and length, and consequently; increased the plant access to plant nutrients and water. The stimulation of root hairs growth and lateral roots elongation by IAA might provide more active sites for nutrient and water absorption and access for interaction with the bacteria to improve root architecture, length and dry weight [61]. Moreover; the bacterial IAA loosen the plant root cell wall and there by facilitates release of excess root exudates which could provide the bacteria with additional nutrients [62]. Therefore, the phyto-beneficial mechanisms of plant growth stimulation like enhanced nutrient availability, phytohormone modulation, biocontrol, biotic and abiotic stress tolerance are exerted by different microbial players [63, 64]. The shoot length and dry weight improvement can be explained by improved nutrient availability, in which phosphorous leads vertical growth and increased biomass. Therefore, this leads to improved nutrient uptake which again leads to enhanced photosynthesis which again demonstrated by increased plant biomass accumulation and productivity [33].

## Conclusion

In this study, we noted that *E. brucei* root nodules harbored diverse types of endophytic bacteria endowed with multiple plant growth promoting traits. These isolated, identified and evaluated root nodule endophytic bacteria exhibited potential of solubilizing sparingly soluble P sources dominated in alkaline and acidic soils. The strains AU4, RG5 and RG6 (*Acinetobacter soli*) are potential candidates for bioinoculant production to enhance growth and productivity of E. brucei both in alkaline and acidic soils in Ethiopia and these strains can also be applied to enrich the plant biomass with phosphorous and these enriched plant biomass can be used as green manure and mulching material as low cost microbial inputs in sustainable crop production. 

## Supplementary Information

**Additional file 1:** 

**Additional file 2:** 

**Additional file 3:**

## Data Availability

The datasets generated and/or analysed during the current study are available in the NCBI database under accession number MK370558 to MK370566.
