# Supplementary material for: Evaluation of inorganic phosphate solubilizing efficiency and multiple plant growth promoting properties of endophytic bacteria isolated from root nodules Erythrina brucei
Source: BMC Microbiol. 2022 Nov 19;22:276. doi: 10.1186/s12866-022-02688-7 (PMC9675159; doi:10.1186/s12866-022-02688-7)
Supplement: Supplementary file 1 — Additional file 1: [file 12866_2022_2688_MOESM1_ESM.docx]

**Supplementary figure 1** Some of the bacterial isolates exhibited clear halos around their colonies on Pikovskaya agar medium supplemented with tricalcium phosphate and incubated for five days at 28°C.
