# Supplementary material for: Evaluation of inorganic phosphate solubilizing efficiency and multiple plant growth promoting properties of endophytic bacteria isolated from root nodules Erythrina brucei
Source: BMC Microbiol. 2022 Nov 19;22:276. doi: 10.1186/s12866-022-02688-7 (PMC9675159; doi:10.1186/s12866-022-02688-7)
Supplement: Supplementary file 2 — Additional file 2: [file 12866_2022_2688_MOESM2_ESM.docx]

| Serial Number | Isolate Designation | Isolate origin /sampling Site | Phosphate solubilization index(SI) | IAA  production | | remark |
| --- | --- | --- | --- | --- | --- | --- |
| 1 | AU1 | AAU | 2.0 | - | |  |
| 2 | AU2 | AAU | 3.0 | + | |  |
| 3 | AU3 | AAU | 1.2 | + | |  |
| 4 | AU4 | AAU | 6.0 | + | |  |
| 5 | AU5 | AAU | 2.5 | + | |  |
| 6 | AU6 | AAU | 3.0 | - | |  |
| 7 | AU7 | AAU | 2.5 | - | |  |
| 8 | AU8 | AAU | 1.5 | + | |  |
| 9 | AU9 | AAU | 2.5 | + | |  |
| 10 | AU10 | AAU | 2.0 | - | |  |
| 11 | AU11 | AAU | 1.5 | - | |  |
| 12 | AU12 | AAU | 2.5 | - | |  |
| 13 | AU13 | AAU | 2.0 | + | |  |
| 14 | AU14 | AAU | 2.0 | - | |  |
| 15 | AU15 | AAU | 1.5 | - | |  |
| 16 | AU16 | AAU | 1.5 | - | |  |
| 17 | AU17 | AAU | 1.0 | + | |  |
| 18 | AU18 | AAU | 1.0 | - | |  |
| 19 | AU19 | AAU | 1.5 | - | |  |
| 20 | AU20 | AAU | 0.85 | - | |  |
| 21 | AU21 | AAU | 0.75 | + | |  |
| 22 | AU22 | AAU | 0.5 | + | |  |
| 23 | AU23 | AAU | 3.57 | - | |  |
| 24 | AU24 | AAU | 3.14 | - | |  |
| 25 | AU25 | AAU | 3.0 | - | |  |
| 26 | AU26 | AAU | 1.0 | + | |  |
| 27 | AU27 | AAU | 1.0 | - | |  |
| 28 | AU28 | AAU | 1.5 | - | |  |
| 29 | AU29 | AAU | 1.5 | - | |  |
| 30 | AU30 | AAU | 2.0 | + | |  |
| 31 | AU31 | AAU | 2.0 | - | |  |
| 32 | AU32 | AAU | 2.5 | - | |  |
| 33 | AU33 | AAU | 2.5 | + | |  |
| 34 | BO1 | Bodit | 2.5 | + | |  |
| 35 | BO2 | Bodit | 2.5 | + | |  |
| 36 | BO3 | Bodit | 3.0 | - | |  |
| 37 | BO4 | Bodit | 3.0 | - | |  |
| 38 | BO5 | Bodit | 1.5 | - | |  |
| 39 | BO6 | Bodit | 2.5 | + | |  |
| 39 | BO7 | Bodit | 1.5 | - | |  |
| 40 | BO8 | Bodit | 1.5 | - |  | |
| 41 | BO9 | Bodit | 2.5 | + |  | |
| 42 | BO10 | Bodit | 1.5 | - |  | |
| 43 | BO11 | Bodit | 1.15 | - |  | |
| 44 | BO12 | Bodit | 2.2 | - |  | |
| 45 | BO13 | Bodit | 1.0 | - |  | |
| 46 | BO14 | Bodit | 2.0 | + |  | |
| 47 | BO15 | Bodit | 1.75 | - |  | |
| 48 | BO16 | Bodit | 1.5 | - |  | |
| 49 | BO17 | Bodit | 2.5 | + |  | |
| 50 | BO18 | Bodit | 3.57 | - |  | |
| 51 | BO19 | Bodit | 3.33 | - |  | |
| 52 | BO20 | Bodit | 1.5 | - |  | |
| 53 | BO21 | Bodit | 2.5 | + |  | |
| 54 | BO22 | Bodit | 2.5 | - |  | |
| 55 | BO23 | Bodit | 2.0 | - |  | |
| 56 | BO24 | Bodit | 2.0 | - |  | |
| 57 | BO25 | Bodit | 1.5 | + |  | |
| 58 | BO26 | Bodit | 1.5 | - |  | |
| 59 | BO27 | Bodit | 1.0 | - |  | |
| 60 | BO28 | Bodit | 2.5 | + |  | |
| 61 | BO29 | Bodit | 3.0 | - |  | |
| 62 | BO30 | Bodit | 3.57 | - |  | |
| 63 | BO31 | Bodit | 3.16 | + |  | |
| 64 | BO32 | Bodit | 1.6 | + |  | |
| 65 | BO33 | Bodit | 2.3 | - |  | |
| 66 | BO34 | Bodit | 1.5 | - |  | |
| 67 | BO35 | Bodit | 2.5 | - |  | |
| 68 | BU1 | Burie | 2.0 | + |  | |
| 69 | BU2 | Burie | 4.12 | - |  | |
| 70 | DM1 | Debremarkos | 1.5 | + |  | |
| 71 | DM2 | Debremarkos | 1.5 | + |  | |
| 72 | DM3 | Debremarkos | 3.0 | - |  | |
| 73 | DM4 | Debremarkos | 2.0 | - |  | |
| 74 | DM5 | Debremarkos | 2.5 | - |  | |
| 75 | DM6 | Debremarkos | 3.25 | + |  | |
| 76 | DM7 | Debremarkos | 2.5 | - |  | |
| 77 | DM8 | Debremarkos | 2.5 | + |  | |
| 78 | DM9 | Debremarkos | 2.0 | - |  | |
| 79 | DM10 | Debremarkos | 1.5 | + |  | |
| 80 | DM11 | Debremarkos | 0.75 | - |  | |
| 81 | DM12 | Debremarkos | 1.0 | + |  | |
| 82 | DM13 | Debremarkos | 2.5 | - |  | |
| 83 | DM14 | Debremarkos | 1.5 | - |  | |
| 84 | DM15 | Debremarkos | 3.0 | + |  | |
| 85 | DM16 | Debremarkos | 3.12 | - | |  |
| 86 | DM17 | Debremarkos | 5.0 | + | |  |
| 87 | DM18 | Debremarkos | 2.5 | - | |  |
| 88 | EN1 | Enrata | 3.33 | + | |  |
| 89 | EN2 | Enrata | 3.57 | - | |  |
| 90 | EN3 | Enrata | 1.5 | - | |  |
| 91 | EN4 | Enrata | 2.0 | - | |  |
| 92 | EN5 | Enrata | 4.2 | + | |  |
| 93 | EN6 | Enrata | 4.43 | + | |  |
| 94 | HO1 | Hadiya | 1.5 | - | |  |
| 95 | HU1 | Humbo | 2.5 | - | |  |
| 96 | HU2 | Humbo | 1.5 | - | |  |
| 97 | IN1 | Injibara | 3.0 | - | |  |
| 98 | IN2 | Injibara | 2.0 | - | |  |
| 99 | IN3 | Injibara | 2.5 | + | |  |
| 100 | IN4 | Injibara | 3.33 | - | |  |
| 101 | GH1 | Gidole | 3.6 | - | |  |
| 102 | GH2 | Gidole | 3.57 | + | |  |
| 103 | GH3 | Gidole | 2.5 | - | |  |
| 104 | GH4 | Gidole | 2.0 | - | |  |
| 105 | GH5 | Gidole | 1.5 | + | |  |
| 106 | GH6 | Gidole | 5.4 | - | |  |
| 107 | GH10 | Gidole | 1.5 | + | |  |
| 108 | RG1 | Rebu Gebeya | 2.5 | + | |  |
| 109 | RG2 | Rebu Gebeya | 2.5 | + | |  |
| 110 | RG3 | Rebu Gebeya | 1.6 | - | |  |
| 111 | RG4 | Rebu Gebeya | 1.5 | - | |  |
| 112 | RG5 | Rebu Gebeya | 4.0 | + | |  |
| 113 | RG6 | Rebu Gebeya | 4.33 | + | |  |
| 114 | TL1 | Tilil | 1.5 | + | |  |
| 115 | TL2 | Tilil | 2.0 | - | |  |
| 116 | TL3 | Tilil | 4.3 | + | |  |
| 117 | TL4 | Tilil | 2.0 | - | |  |
| 118 | TL5 | Tilil | 1.5 | + | |  |
| 119 | TL6 | Tilil | 2.5 | - | |  |
