# Supplementary material for: Evaluation of inorganic phosphate solubilizing efficiency and multiple plant growth promoting properties of endophytic bacteria isolated from root nodules Erythrina brucei
Source: BMC Microbiol. 2022 Nov 19;22:276. doi: 10.1186/s12866-022-02688-7 (PMC9675159; doi:10.1186/s12866-022-02688-7)
Supplement: Supplementary file 3 — Additional file 3: [file 12866_2022_2688_MOESM3_ESM.docx]

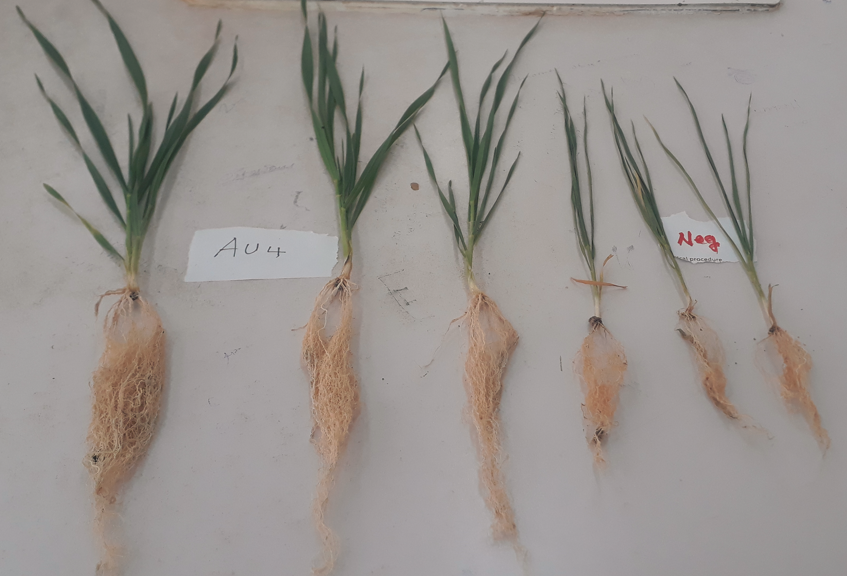


Fig.2 A wheat plant growth parameters AU4 inoculated plant against negative control


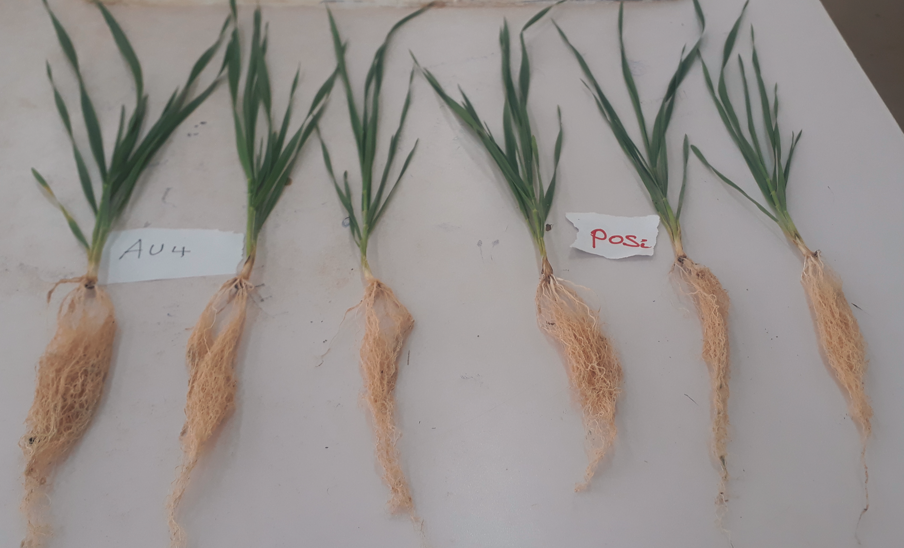


Fig.2B wheat plant growth parameters AU4 inoculated plant against positive control


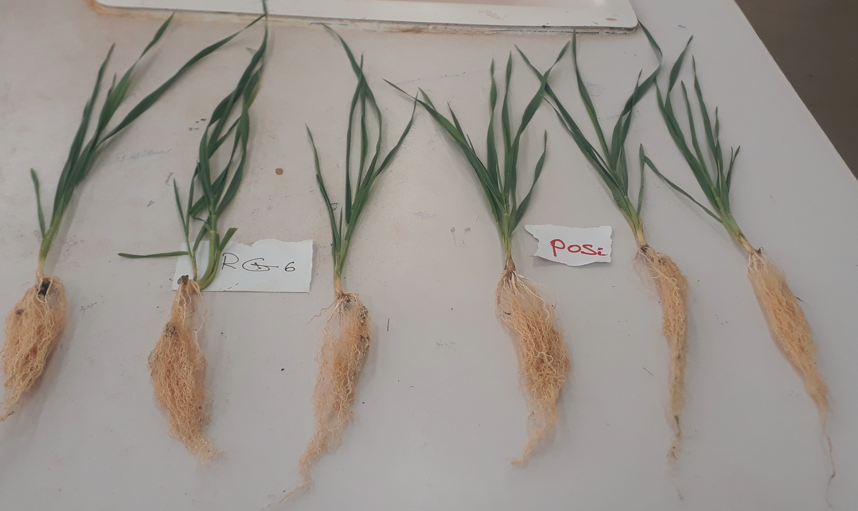


Fig.2C wheat plant growth parameters RG6 inoculated plant against positive control


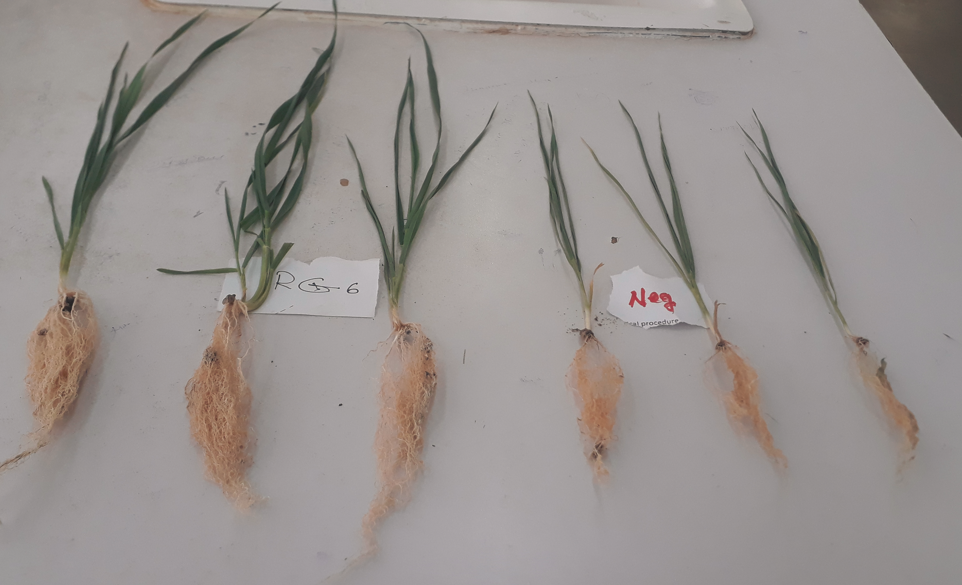


Fig.2D wheat plant growth parameters RG6 inoculated plant against negative control


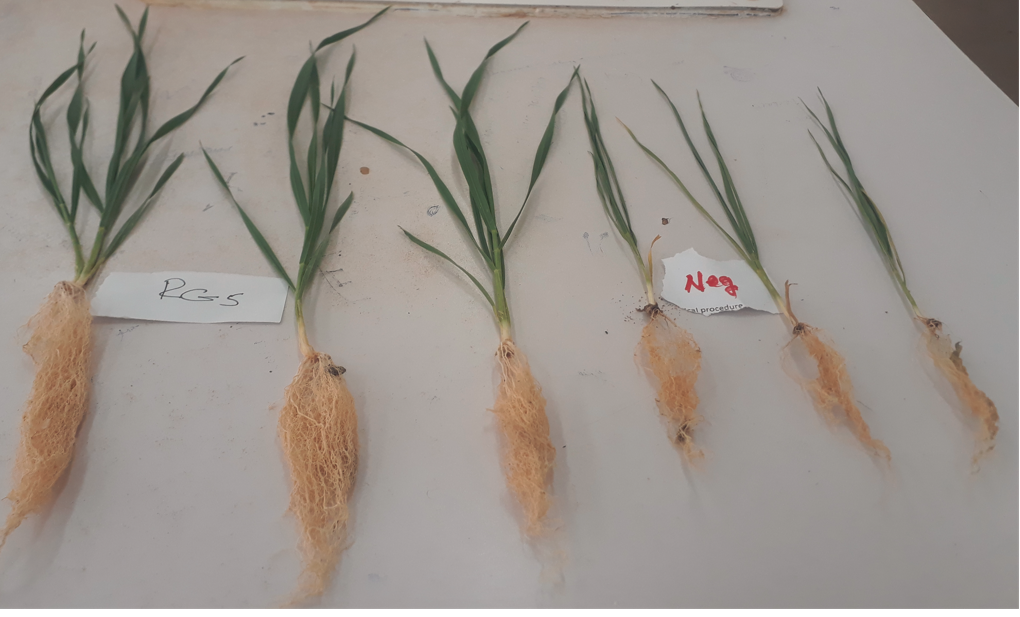


Fig.2E wheat plant growth parameters RG5 inoculated plant against negative control


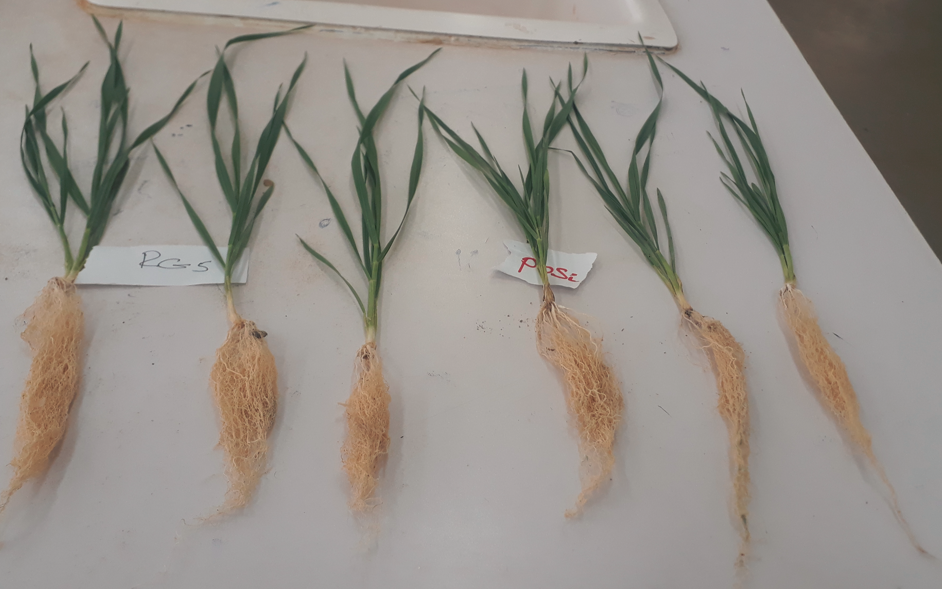


Fig.2F wheat plant growth parameters RG5 inoculated plant against positive control
